# *Dosis Facit Sanitatem—*Concentration-Dependent Effects of Resveratrol on Mitochondria

**DOI:** 10.3390/nu9101117

**Published:** 2017-10-13

**Authors:** Corina T. Madreiter-Sokolowski, Armin A. Sokolowski, Wolfgang F. Graier

**Affiliations:** 1 Institute of Molecular Biology and Biochemistry, Medical University of Graz, Neue Stiftingtalstraße 6/6, 8010 Graz, Austria; wolfgang.graier@medunigraz.at; 2 Department of Dentistry and Maxillofacial Surgery, Medical University of Graz, Billrothgasse 4, 8010 Graz, Austria; armin.sokolowski@medunigraz.at

**Keywords:** resveratrol, mitochondria, concentration-dependent effects, cytotoxic agent, cytoprotection

## Abstract

The naturally occurring polyphenol, resveratrol (RSV), is known for a broad range of actions. These include a positive impact on lifespan and health, but also pro-apoptotic anti-cancer properties. Interestingly, cell culture experiments have revealed a strong impact of RSV on mitochondrial function. The compound was demonstrated to affect mitochondrial respiration, structure and mass of mitochondria as well as mitochondrial membrane potential and, ultimately, mitochondria-associated cell death pathways. Notably, the mitochondrial effects of RSV show a very strict and remarkable concentration dependency: At low concentrations, RSV (<50 μM) fosters cellular antioxidant defense mechanisms, activates AMP-activated protein kinase (AMPK)- and sirtuin 1 (SIRT1)-linked pathways and enhances mitochondrial network formation. These mechanisms crucially contribute to the cytoprotective effects of RSV against toxins and disease-related damage, in vitro and in vivo. However, at higher concentrations, RSV (>50 μM) triggers changes in (sub-)cellular Ca^2+^ homeostasis, disruption of mitochondrial membrane potential and activation of caspases selectively yielding apoptotic cancer cell death, in vitro and in vivo. In this review, we discuss the promising therapeutic potential of RSV, which is most probably related to the compound’s concentration-dependent manipulation of mitochondrial function and structure.

## 1. Introduction

Nature has always been a valuable source for medicine. Notably, many currently-used indispensable drugs were originally developed from plant-derived compounds, such as the antihypertensive agent, verapamil, which is based on papaverine from the opium poppy (*Papaver somniferum*), or the antidiabetic agent metformin, based on the substance, galegine, from French lilacs (*Galega officinalis*) [1]. In the last decades, driven by the compounds’ beneficial effects on human health, a special interest in polyphenols has occurred. This huge, diverse group of plant metabolites includes well-known compounds, such as the phytoalexin, resveratrol (3,4′,5-trihydroxy-*trans*-stilbene; RSV) and the flavonoid, quercetin [2]. The actions of RSV have been studied in several in vivo and in vitro approaches, demonstrating antioxidant, anti-inflammatory and anti-aging effects [3], but also pro-apoptotic anti-cancer properties [4,5] as well as antimicrobial activity [6].

Since RSV is produced by plants to fight against microbes [1], it seems reasonable that this specific compound also strongly affects mitochondria, which are thought to be descendants from ancient bacteria that have been incorporated as an endosymbiont into an ancestor in the modern eukaryotic cell [2,3]. By hosting the complexes of the respiration chain in their inner membranes (IMM), mitochondria are the predominant source for the cell’s most important energy carrier, adenosine triphosphate (ATP). Consequently, the control of mitochondrial activity is of utmost importance for cellular wellbeing [4] and, obviously also for the therapeutic action of RSV, as experiments in mitochondrial DNA deficient Rho 0 cells have revealed functional mitochondria to be crucial for the effects of RSV to occur [5]. Interestingly, experiments revealed a strong concentration-dependent impact of RSV on mitochondrial function, which is of major importance, due to the therapeutic potential of this polyphenol [6,7,8]. This review provides an overview of these findings and intends to estimate the medical potential of mitochondrial manipulation by RSV.

## 2. Mitochondria—Structure and Function

Mitochondria are very dynamic, double-membraned organelles in eukaryotic cells [4]. They continuously undergo structural changes, like fusion and fission, to ensure adaptation to various cellular conditions [9]. The outer mitochondrial membrane (OMM) separates the cytosol from the intermembrane space (IMS) and only allows diffusion of small molecules, up to a size of approximately 6 kDa [10]. The inner mitochondrial membrae (IMM) is practically impermeable. Therefore, specific (protein) transporters exist in the OMM (e.g., translocases of the outer membranes, TOMs) and the IMM (e.g., translocases of the inner membranes, TIMs) [11]. Hence, the IMM harbors specific small molecule carriers like the ADP/ATP translocase, the carnitine/acylcarnitine carriers [12], the complexes of the respiration chain [13], the mitochondrial Ca^2+^ uniporter [14] and the mitochondrial Na^+^/Ca^2+^ exchanger [15]. The surface area of the IMM is strongly increased by numerous infoldings (i.e., cristae), stretching deeply into the mitochondrial matrix and hosting proteins of the mitochondrial respiration chain, which is responsible for the cell’s most efficient production of ATP, by oxidative phosphorylation (OXPHOS) [4].

On average, a human requires approximately 420 kJ per hour during resting conditions, which corresponds with the production of 65 kg of ATP per day. This high demand for ATP represents a Herculean task for the mitochondria, which transfer electrons from nicotinamide adenine dinucleotide (NADH/H+)^+^ and flavin adenine dinucleotide (FADH_2_) to the final electron acceptor, oxygen, to form water, within the electron transport chain (ETC) [16]. The ETC consists of NADH dehydrogenase (complex I), succinate dehydrogenase (complex II), ubiquinone, bc1-complex (complex III), cytochrome c (cyt c), and cytochrome c oxidase (complex IV). Electron transport along these proteins is coupled to proton pumping by complexes I, III, and IV, from the mitochondrial matrix, through the IMM into the IMS. This results in an electrochemical gradient, which serves as energetic drive for the F_o_F_1_ ATP synthase that utilizes the energy gained during the reflux of the protons to phosphorylate adenosine diphosphate (ADP) to ATP in the mitochondrial matrix [17]. In case of incomplete electron transfer (also called electron leakage) at complex I and complex III, superoxide anions are produced as by-products of the ETC. These reactive oxygen species (ROS) are converted by mitochondrial superoxide dismutase 2 (SOD2, MnSOD) to H_2_O_2_ [18].

Since mitochondria are essentially involved not just in energy supply [17,19], but also in cell differentiation [20], cell cycle control [21], signal transduction [22] and Ca^2+^ homeostasis [23], they are very promising potential drug targets for treating various diseases, including neurodegenerative [24] and cardiovascular diseases [25,26] and cancer [27,28].

## 3. Resveratrol—Sources and Bioavailability

RSV was first isolated in 1939, from the roots of the white hellebore and is also found in several other plants, including grapes, apples, blueberries, pistachios and peanuts [29]. The research on RSV was greatly boosted by the detection of the so-called “French paradox” in the beginning of the 1990s. This phenomenon describes the low incidence of coronary heart diseases in French people despite their diet, which is relatively rich in saturated fatty acids that are well-known risk factors for atherosclerosis and coronary heart disease [30]. Notably, the common consumption of red wine was proposed to account for the protection of the French people. Therefore, the chemical composition of red wine has been intensively studied and has been found to be a rich source for RSV (up to 6.8 mg/L), thus, bringing this compound into the spotlight of medical research [31,32]. Notably, RSV exists naturally as *cis*- and predominantly as *trans*-isomers. Due to higher chemical stability, most studies use the *trans*-RSV [33].

The absorption and bioavailability of orally administered (*p.o.*) 25 mg *trans*-RSV, estimated as an equivalent amount to moderate red wine intake, was studied in healthy humans [34,35]. The small intestine was found to absorb more than 70% of the RSV, probably due to the small and non-polar characteristics of *trans*-RSV, which may allow absorption across membranes by passive diffusion [36] or active transport via the intestinal epithelium via ATP-dependent binding cassette (ABC) transporters [37]. However, due to extensive metabolism in the intestine and liver, the bioavailability of orally administered RSV is very low [37]. After oral administration of 25 mg, *trans*-RSV peak plasma levels of RSV metabolites reached 500 ng/mL (approx. 2 μM) after 30 min, while only trace amounts of unchanged RSV could be detected (<5 ng/mL) [35]. These studies point to a discrepancy between the concentrations of RSV in in vitro experiments, where the values of the half maximal effective concentration (EC_50_) range from 1 to 100 μM and the actual plasma concentrations are a maximum of 40 nM of unmetabolized RSV [34]. Dose-escalating studies, involving an oral dose of up to 5 g RSV in healthy volunteers [38] and up to 1 g in Alzheimer’s patients [39] revealed that even high-dose *trans*-RSV *p.o.* caused just a small peak in plasma levels of unchanged RSV (approx. 2 μM), whereas the peak levels of the glucuronides and sulphate metabolites were 2- to 8-fold higher [38].

Another study investigated the effects of 500 mg RSV tablets, which are available on the market, and described that the total plasma level of RSV and metabolites reaches approximately 5 μM, a concentration shown to be active in cell culture experiments [37]. Since the most frequently detected metabolites of RSV—glucuronides and sulphates [40]—have pharmacologically similar activity to RSV, it is tempting to speculate that the metabolites of RSV contribute to the biological effects of RSV in vivo. Notably, a multiple-dose study in healthy humans supported this conclusion, but revealed a large inter-individual variability in peak plasma concentrations as well as circadian variations [41].

Taken together, conforming studies report that oral administration of ≥0.5 g RSV leads to therapeutically considerable levels in human plasma and the gastrointestinal tract that may exhibit cytoprotective and anticarcinogenic effects, respectively (see paragraph “*Duality in effects of resveratrol*” below) [42]. Accordingly, topical application of RSV protected mice from skin tumor formation and development [43]. For systemic cancer treatment, new formulation strategies have to be found to overcome RSV’s poor bioavailability and to reach cytotoxic levels of this compound [44]. In this respect, an enhanced systemic availability of RSV was reported by applying a dual nano-encapsulation approach [45], thus, giving hope that solutions might be soon available to circumvent the low bioavailability of RSV.

## 4. Mitochondrial Targets of Resveratrol

### 4.1. Mitochondrial Respiration

Manipulations of the mitochondrial respiration chain strongly affect cellular homeostasis and function, due to mitochondria’s crucial role in ATP supply [46]. Notably, RSV itself targets several complexes of the mitochondrial respiration chain (Table 1).

Complex I: At low concentrations (1–5 μM for 48 h) RSV stimulates complex I in the immortalized human hepatoblastoma cell line (HepG2) causing an increased NAD^+^/NADH ratio and enhancing mitochondrial supply pathways, via mitochondrial sirtuin (SIRT3) [47]. In line with this effect, RSV was reported to enhance complex I activity in brain mitochondria [48] and to cause increased mitochondrial respiration and NAD^+^/NADH ratios in liver mitochondria of mice fed with an RSV-enriched diet (40 mg/kg/day or 50 mg/kg/day) for 12 weeks [47]. Due to the low bioavailability of RSV, these doses resulted in low peak plasma levels, correlating with the low dose RSV used in cell culture experiments. In contrast, high concentrations of RSV (>50 μM) resulted in decreased activity of complex I in HepG2 cells [47].

Complex III: In addition to complex I, another main production site of mitochondrial ROS, complex III [61], has been shown to be affected by RSV [61]. Accordingly, treatment of isolated mitochondria from rat brains with 100 μM RSV caused an inhibitory effect on complex III [7].

F_o_F_1_ ATP synthase: RSV was also proven to bind to the F_1_ subunit of F_o_F_1_ ATP synthase [62,53]. In mitochondrial fractions of rat livers, very low doses of RSV (in the pM–nM range) caused activation of F_o_F_1_ ATP synthase [52], whereas higher doses caused inhibition of this enzyme in mitochondrial fractions of liver (IC_50_ = approx. 12.0 μM) [52,53,62], brain (IC_50_ = 18.5 μM) [62,53], and heart (IC_50_ = 12–15 μM) [52].

It seems contradictory that low concentrations of RSV trigger, and high concentrations inhibit, respiratory chain complexes. In view of the present data, we assume that slight inhibition of these proteins by low RSV concentrations leads to compensatory mechanisms, such as upregulation, ending up with a measurable increase in activity of these complexes. Nevertheless, this compensation might not be sufficient to overcome the inhibitory effect of RSV at higher concentrations.

ATP and ROS production: Live-cell imaging of HeLa (human cervical cancer line) cells has revealed a strong decrease in mitochondrial ATP and a slight drop in cytosolic ATP levels in response to 100 μM RSV, within several minutes [6]. In contrast, treatment with 10 μM RSV for 48 h caused an initial increase in mitochondrial respiration activity and ATP levels, followed by hyperpolarization of the mitochondrial membrane, increased ROS production and apoptosis in colon carcinoma cells (SW620) [49]. In fibroblasts of patients with complex I (CI) deficiencies, even rather high levels of RSV (75 μM, 48 h) increased the amount of cellular ATP and decreased intracellular ROS levels, by enhanced ROS defenses, such as elevated SOD2 levels [50]. In line with this, RSV treatment (250 mg/kg/day) for 3 weeks increased ATP levels of wild-type and cuprizone-intoxicated mice and enhanced SOD activity and GSH levels [54]. Furthermore, RSV also enhanced mitochondrial enzyme activity and oxidative capacity in muscles of RSV-fed mice, attenuating the sensitivity of animals to diet-induced obesity and insulin resistance [51]. Interestingly, in an Alzheimer’s disease model, pretreatment with 5 μM of RSV for 6 h scavenged beta amyloid-triggered alterations in the mitochondrial structure of mouse neuroblastoma cells (neuro-2A), but failed to reduce elevated H_2_O_2_ production [63]. Notably, the ubiquinone derivative, MitoQ, which acts as an electron scavenger and thereby prevents mitochondrial ROS formation [64], could normalize H_2_O_2_ levels under this condition [63]. This finding might indicate that mitochondrial-targeted antioxidants are more effective than RSV in preventing ROS-related cellular damage. In addition, RSV’s mode of action might require the ability to form a ROS defense shield, a mechanism potentially hampered in the case of already high-grade cellular damage. For instance, brain mitochondria from old mice with low antioxidant defenses, displayed enhanced oxidative stress after RSV treatment, whereas the same doses of RSV did not cause an increase in oxidative damage in young mice [48]. In *Caenorhabditis elegans* (*C. elegans*) RSV significantly attenuated oxidative stress and prolonged life span [65]. Accordingly, individual ability to develop defense mechanisms against ROS might be crucial for the physiological effects of administration of RSV.

### 4.2. Mitochondrial Mass and Structure

Mitochondrial mass and network structure, defined by fusion and fission, are essential for mitochondrial function [66]. Several reports have described considerable changes in mitochondrial mass and network formation, as a result of RSV treatment (Table 1). For instance, treatment with 10 μM of RSV for 48 h resulted in a significant increase in mitochondrial number and mitochondrial DNA content in human umbilical vein endothelial cells (HUVECs), probably due to RSV-triggered sirtuin 1 (SIRT1) activity and induction of mitochondrial biogenesis factors, such as peroxisome proliferator-activated receptor-gamma coactivator 1-α (PGC1α), mitochondrial transcription factor A (Tfam) and nuclear respiratory factor-1 (Nrf-1) [57]. This is in line with the effects of RSV in human coronary arterial endothelial cells (CAECs) where RSV augments the expression of mitochondrial biogenesis factors—PGC1-α, Tfam and Nrf-1—leading to increased mitochondrial mass and mitochondrial DNA content. Notably, all these effects have been prevented by knockdown of SIRT1, pointing to the crucial role of SIRT1 in RSV-triggered mitochondrial biogenesis. This finding was further strengthened by a normalization of impaired mitochondrial biogenesis in aortas of diabetic (db/db) mice after *p.o.* administration of 20 mg/kg/day RSV for 4 weeks [58]. Moreover, muscles of mice fed with 200–400 mg/kg/day *p.o*. for 15 weeks (corresponding to plasma levels of 10–120 ng/mL [40 nM–500 nM]), displayed increased mitochondrial size and density as well as enhanced levels of mitochondrial DNA. Again, activation of PGC1-α by SIRT1-driven deacetylation was crucial for the effect of RSV [51]. In the colon carcinoma cell line, SW620, treatment with 10 μM of RSV for 48 h induced upregulation of PGC1-α, Tfam and Nrf-1, which resulted in increased mitochondrial mass. Notably, expression of sirtuin 3 (SIRT3), known to activate proteins related to mitochondrial energy metabolism, was strongly enhanced by RSV treatment, too [49]. Furthermore, mitochondrial biogenesis induction has also been confirmed for the immortalized human hepatoblastoma cell line, HepG2, after treatment with 1 μM RSV for 12 h [55].

In contrast, at higher concentrations of RSV (50 μM, 48 h) an induction of autophagy was reported in the human peritoneal mesothelial cell line, HMrSV5 [60]. Another study demonstrated that low levels of RSV (1–10 μM, 24 h) increased mitophagy features in a hepatic stellate cell model (GRX). In regard to this cell model, a strong concentration dependency for RSV’s effect on mitochondrial structure and mass was reported.

Notably, mitochondria constantly undergo fusion and fission to remodel and exchange contents. The proteins, mitofusin 1 (MFN1) and mitofusin 2 (MFN2), mediate OMM tethering and fusion [67], while the process of fission is controlled by mitochondrial fission 1 protein (FIS1) [68] and dynamin-related protein-1 (DRP1); these proteins are recruited to the OMM to form oligomers and divide mitochondria at discrete sites [69]. In parallel, the protein, optic atrophy 1 (OPA1), shapes the IMM structure and cristae morphology [70]. While 24 h treatment with concentrations of 1–10 μM RSV caused an increase in the expression of proteins involved in mitochondrial fusion (MFN1, MFN2, OPA1), the expression of these proteins was reduced by 50 μM of RSV [56]. Accordingly, a crucial contribution of MFN2 to the effect of RSV was demonstrated by highly branched mitochondrial networks in C2C12 myoblasts, in the prostate cancer cell line, P3, and in mouse embryonic fibroblasts (MEFs) after treatment with 10–20 μM RSV for 48 h, due to stimulated MFN2 expression [59]. Furthermore, RSV treatment (10–60 μM, 24 h) protected rotenone-exposed rat pheochromocytoma cells (PC12) from mitochondrial fragmentation and normalized the expression of DRP1, OPA1, MFN2 and FIS1 [71]. Upregulation of MFN2 by RSV treatment (20 μM, 24 h) also suppressed cigarette smoke extract (CSE)-induced mitochondrial dysfunction in human bronchial epithelial cells [72]. Moreover, RSV (50 mg/kg/day, 7 days) was reported to inhibit ROS-associated mitochondrial fission, by upregulation of DRP1, via increased AMPK phosphorylation activity in the adipose tissue of mice with streptozotocin-induced diabetes. This mechanism protected adipose tissue against high glucose-induced injury [73]. All these reports indicate that RSV strongly affects cells through structural changes in mitochondria.

### 4.3. Mitochondrial Apoptotic Pathways

Ca^2+^ homeostasis: In cell culture experiments, RSV at concentrations higher than 50 μM, often displays cytotoxic features, via activation of mitochondrial apoptotic pathways linked to mitochondrial Ca^2+^ overload, disruption of mitochondrial membrane potential and activation of pro-apoptotic caspases (Table 2) [74,75]. Treatment with 100 μM of RSV caused an early increase in free intracellular Ca^2+^ in human breast cancer cell lines—MCF-7 and MDA-MB-213—probably due to Ca^2+^ leakage from the endoplasmic reticulum, which subsequently led to mitochondrial Ca^2+^ overload and a disruption in mitochondrial membrane potential [74]. After long-term treatment with 100 μM of RSV (24–72 h), this process resulted in the release of cytochrome c and mitochondrial-derived caspase activators, caspase activation and stimulation of the Ca^2+^-activated protease, calpain, ultimately leading to apoptosis of breast cancer cells [74]. A similar process was described for RSV-induced apoptosis in HepG2 cells [75]. Subsequent to the elevation in intracellular Ca^2+^ by 100 μM of RSV (2–8 h), the membrane potential collapsed (10–12 h), causing mitochondrial permeability transition pore (mPTP) opening and cytochrome c release into the cytosol [75]. Our group also identified the elevation in mitochondrial Ca^2+^ as a trigger for RSV-induced apoptosis. In HeLa, a human cervical cancer cell line, and EA.hy926, a human hybridoma cell line from primary human umbilical vein cells and the human lung carcinoma cell line, A549, 100 μM of RSV caused a strong decrease in mitochondrial and cytosolic ATP levels, through inhibition of F_o_F_1_ ATP synthase. This RSV-evoked ATP depletion hampers activity of sarco/endoplasmic reticulum Ca^2+^ ATPase (SERCA), which limits Ca^2+^ transfer into mitochondria by re-sequestering junctional Ca^2+^ into the ER within the ER–mitochondria junctions. Consequently, in the presence of RSV inositol, 1,4,5-trisphosphate-generated Ca^2+^ release from the ER yields mitochondrial Ca^2+^ overload in HeLa and EA.hy926, leading to caspase activation, and ultimately, to apoptotic cancer cell death [6]. The importance of SERCA2 and SERCA3 on the effect of RSV has also been demonstrated in the two breast cancer cell lines, MCF7 and MDA-MB-231 [76]. Paradoxically, SERCA3 expression was even increased after 48–72 h of treatment with 50–200 μM RSV in MCF7 and MDA-MB-231 cells, potentially a sign of a cellular adaptation process against the cytotoxic effect on Ca^2+^ homeostasis of RSV [76]. In the prostate cancer cell lines, PC3 and DU145, treatment with 100 μM RSV for 24 h caused decreased ER Ca^2+^ storage capability and reduced store-operated Ca^2+^ entry (SOCE), inducing ER stress and autophagic cell death [77].

Mitochondrial membrane potential: In the uveal melanoma cell lines—M619, C918 and Mum2b—50–200 μM of RSV caused dissipation of the mitochondrial membrane potential after 15 min (Table 2), which was followed by the release of cytochrome c and the proapoptotic proteins (Smac/Diablo), leading to caspase activation and apoptosis after 3–24 h [78]. In a very similar manner, RSV induced apoptotic cell death in several cancer cell lines, including lung cancer cell lines (H383 and H520) [79], renal cell carcinoma cell lines (Caki-1 and 786-O) [80], lung cancer cells (A549) [81], human colorectal carcinoma (HT-29) [82], murine prostate carcinoma cells (TRAMP-C1, TRAMP-C2 and TRAMP-C2) [83] and human prostate cancer cells (LNCaP) [84].

## 5. Opposing Effects of Resveratrol

### 5.1. Cytoprotective Actions of Resveratrol

Protective potential of RSV against toxins: RSV affects the two main sources of mitochondrial ROS production (i.e., complex I and complex III; [61]), and thus, influences cellular ROS production. Low concentrations of RSV have been reported to cause a hormetic effect, through induction of moderate ROS formation via increased activity of the mitochondrial respiration chain, which triggers upregulation of antioxidant defenses [85]. Consequently, RSV is able to display its cytoprotective abilities (Table 3) against various toxins. The RSV-induced protection also includes elevated expression of enzymes of the cellular ROS defense, including mitochondrial MnSOD, cytosolic/nuclear Cu/ZnSOD, catalase (CAT), glutathione reductase (GR) and glutathione peroxidase (GPx), and downregulation of the ROS-producing enzymes, like NAPDH oxidases 1 and 2 (NOX-1, NOX-2) [86]. It was also demonstrated that 24 h of pre-treatment of CHO-K1 cells with low concentrations of RSV (2.5–5 μM) prevents ROS production and cell death induced by beauvericin, a contaminant of cereals [87]. In Bhas 42 fibroblasts, 2 h of 5 μM RSV treatment inhibited oxidative stress and neoplastic transformation induced by benzo[a]pyrene, a carcinogenic compound found in cigarette smoke and grilled food [88]. In addition, a cytoprotective effect of RSV (12.5 μM, 48 h) due to a reduction in oxidative stress was reported in the human renal cell line, HK-2, that was exposed to the toxic radiocontrast agent, ioxitalamat [89]. In line with this, 24 h of treatment with 20 μM of RSV caused protection by triggering MnSOD activity in renal proximal tubule cells (NRK52E) facing nicotine-triggered oxidative injury [90]. Notably, in primary astrocytes of rats, rather high concentrations of RSV (100 μM, 1 h) prevented azide-induced mitochondrial dysfunction, by initiating ROS defense mechanisms and modulation of pathways involving p38 mitogen-activated protein kinase (p38 MAPK) and nuclear factor kappa B (NFkB) [91,92], indicating that various cell types exhibit different susceptibilities to RSV’s effects, as presented in a review by de Oliveira et al. [8].

Another mechanism of RSV-initiated cell-protection was described in human bronchial epithelial (HBE) cells. In this cell type, RSV (20 μM for 2 h) prevented cell death, induced by cigarette smoke extract, via increasing MFN2 levels and preventing mitochondrial membrane potential loss and cytochrome c release [72].

In RAW 264.7 macrophages, stressed with 2,2′-azobis(2-amidinopropane) dihydrochloride (AAPH), RSV-triggered (2.5 μM, 24 h) autophagy was promoted by upregulation of SIRT3 and activation of AMP-activated protein kinase (AMPK) [94]. This protein is crucially involved in the regulation of cellular energy homeostasis and the glucose restriction-mediated life span increase, via mitohormesis, a mechanism initiating health-promoting antioxidant defense mechanisms by low levels of ROS [101]. Moreover, RSV also prevented sepsis-induced injury to organs and organelles, by modulating mitochondrial function [102].

Protective potential of RSV in human disease: Cardiovascular and metabolic diseases: Extensive ROS production occurs not just on chemicals but also in many diseases, such as cardiovascular and metabolic diseases. Therefore, development of ROS defense mechanisms induced by RSV at low levels might be the key to preventing cellular damage in the cardiovascular and metabolic systems. For instance, in coronary aortic endothelial cells (CAECs), 24 h of treatment with 10 μM RSV caused an increase in antioxidative potential via enhanced expression of MnSOD and elevation of glutathione (GSH) levels [58]. Low levels of RSV (10 μM, 1 h) also attenuated oxygen glucose deprivation/reoxygenation (OGD/R)-induced apoptotic cell damage in rat glioma cells (P12) and increased MnSOD and CAT activities [95]. Moreover, a diet enriched with RSV (100 mg/kg per day, 3 months) enhanced the expression of SERCA2a, which has been shown to play a crucial role in cardiac dysfunction [103], causing an improvement in cardiac function. Notably, this effect was absent in SIRT1 knockout mice, highlighting the crucial role of SIRT1 in conveying RSV’s effects [100]. RSV was also shown to alleviate diabetic cardiomyopathy in rats, throughPGC1-α deactylation by SIRT1, leading to an increased mitochondria DNA copy number and elevated ATP levels. Moreover, activity of MnSOD was elevated in this diabetic animal model by RSV administration (50 mg/kg/day, 16 weeks) [98].

Another protective mechanism, triggered by RSV, was demonstrated to work via AMPK. In the renal fibroblast cells (NRK-49F), treatment with 10–20 μM RSV for 1 h yielded enhanced AMPK activity. In vivo investigations revealed increased active phospho-AMPK in the kidneys of diabetic (db/db) mice treated with RSV (40 mg/kg/day, 12 weeks). Elevated AMPK activity caused a decrease in NOX4 expression and in its associated ROS production, which attenuated renal fibrosis [96]. Moreover, RSV (50 mg/kg/day, 7 days) was reported to inhibit ROS-associated mitochondrial fission by upregulating DRP1 phosphorylation via increased AMPK activity in the adipose tissue of mice with streptozotocin-induced diabetes. This mechanism protected adipose tissue against high glucose-induced injury [73]. AMPK activity was also strongly enhanced in H9c2 muscle cells by 50 μM RSV. In parallel, ROS levels decreased and H_2_O_2_-induced cell death, mimicking ischemic conditions, was strongly attenuated [97]. All these beneficial effects make RSV a promising drug for the future treatment of cardiovascular diseases [104,105].

Neurodegenerative diseases: In a mouse model (YAC128) of Huntington’s disease—a neurodegenerative disease strongly linked to mitochondrial dysfunction—continuous RSV treatment for 28 days (1 mg/kg per day s.c.) restored the expression of mitochondrial-encoded electron transport chain proteins and significantly improved motor coordination and learning abilities [99]. Hence, AMPK-linked protection mechanisms induced by RSV are reported as crucial in the treatment of neurodegenerative diseases [99]. Administration of a daily RSV-enriched diet (160 mg/kg RSV) significantly delayed the onset of amyotrophic lateral sclerosis (ALS) in a SOD1^G93^A mouse model, by improving spinal motoneuron function, extending survival through the activation of SIRT1 and AMPK and promoting mitochondrial biogenesis in the spinal cord [99]. In line with this, it was shown that treatment of neuronal Neuro2a cells with 10 μM of RSV for 2 h caused mitochondrial biogenesis via AMPK-activation, by mimicking caloric restriction [106]. Moreover, pre-treatment for 24 h with very low levels of RSV (<75 nM) rescued mitochondrial dysfunction induced by the neurotoxin, 1-methyl-4-phenylpyridinium (MPP+), known to provoke Parkinson’s disease-like symptoms, through an AKT/GSK-3b pathway in the dopaminergic neuron cell line, SN4741 [93]. Several other effects of RSV on brain mitochondria were reviewed just recently by Jardim et al. [92].

In line with neurodegenerative diseases, a recent study on fibroblasts of patients suffering from severe mitochondrial diseases [107] has revealed that low doses of RSV markedly improve mitochondrial function and cell viability [108], which makes this compound and its mode of action especially interesting for further investigations.

Aging: RSV has gained great attention for its lifespan extending effects in *Saccharomyces cerevisiae* [109], *C. elegans* [110], *Drosophila melanogaster* (*D. melanogaster*) [111], fish [112] and mice [113]. Thereby, the activation of SIRT1 represents a hallmark in the lifespan extending effect of RSV [99,106]. This deacetylase has been shown to enhance mitochondrial biogenesis [114] and metabolism [115] as well as to extend lifespan in yeast [116], worms [117] and flies [118]. However, the hypothesis that SIRT1 is the key mediator of RSV’s effect on lifespan was challenged by a report demonstrating that the increased lifespan of *C. elegans* and *D. melanogaster* after RSV treatment was preserved in strains lacking the functional SIRT1 orthologue, Sir2 [119]. Moreover, several reports contradict the direct activation by RSV of SIRT1 [120,121,122] and suggest an indirect stimulation of SIRT1 by AMPK, via an increase in NAD^+^ levels [123]. As a key protein in controlling energy homeostasis and resistance to stress, AMPK is crucially involved in promoting health and lifespan [124]. Different pathways for stimulation of this enzyme by RSV are feasible. At high concentrations of RSV (i.e., >50 μM) the inhibition of ATP synthase is speculated to activate AMPK, through increased AMP/ATP and ADP/ATP ratios [125,126]. Nevertheless, studies in murine tissues revealed activation of AMPK by RSV at concentrations of less than 10 μM. Notably, RSV has been shown to mimic features of caloric restriction (CR)—including glucagon and catecholamine release—stimulating adenylylate cyclase and boosting cyclic AMP (cAMP) production. Through cAMP, cell-type dependent effectors might be activated, leading to increased Ca^2+^ levels and activation of the CAMKKβ-AMPK pathway [127]. Furthermore, RSV has also been shown to induce low levels of mitochondrial ROS production, which might stimulate AMPK activity as well [128]. The induction of low level mitochondrial ROS production by RSV was also found to cause a mitohormetic response in *C. elegans*, stimulating the development of enhanced antioxidant defense mechanisms [129]. Thereby, RSV might be protective against various toxins and disease-related damages (see paragraph “Cytoprotective actions of resveratrol” above), resulting in an extended lifespan.

### 5.2. Cytotoxic Actions of Resveratrol

The induction of apoptosis by RSV via a mitochondria-dependent pathway, in concentrations >50 μM, was described for various cancer cell types (Table 2). As described in detail in the section “Mitochondrial apoptotic pathways”, RSV treatment with concentrations of >50 μM strongly reduces the cell viability of many human cancer cells, while very low levels of RSV are likely to enhance cancer cell proliferation [130], potentially by mechanisms described in the section “Cytoprotective actions of resveratrol”. Several studies have described an intracellular Ca^2+^ rise as the trigger for the disruption of mitochondrial membrane potential and the initiation of apoptotic caspase activity, finally leading to cell death [6,74,75].

Interestingly, cancer cells have been reported as more vulnerable to the cytotoxic effects of RSV than corresponding non-cancerous cells. For instance, treatment with 50 μM of RSV for 24 h caused apoptosis in human prostate carcinoma (LNCaP) via the loss of mitochondrial membrane potential and an increase in proapoptotic BCL-2 proteins, whereas similar concentrations did not affect normal human prostate epithelial cells [84]. In line with these findings, treatment with 50 μM of RSV triggered cell death in glioma cell lines within 72 h, while primary astrocytes from rats remained largely unaffected [131]. In our previous work, we showed that mitochondrial Ca^2+^ uptake of freshly isolated HUVECs after IP_3_-generating agonist stimulation was unchanged after treatment with 100 μM of RSV, whereas in corresponding cancerous EA.hy926 cells, mitochondrial Ca^2+^ uptake was strongly increased under the same conditions [6]. Accordingly, increased stability in mitochondria-associated ER membranes (MAMs) was found in EA.hy926 and HeLa cells, relative to non-cancerous cells, pointing to an enforced tethering between mitochondria and ER that makes cancer cells more vulnerable for RSV-triggered toxicity [6]. Consequently, screening for MAM stability in various cancer cells might potentially help to identify cancer types susceptible to RSV-induced cell death.

As described above, RSV has been already successfully tested in the treatments of colorectal cancer [42] and skin tumors [43]. For other cancer types, new pharmaceutical formulations are needed to gain full profit from the compound’s cytotoxic effects, by overcoming the compound’s low bioavailability.

## 6. Conclusions

**Scheme 1:**
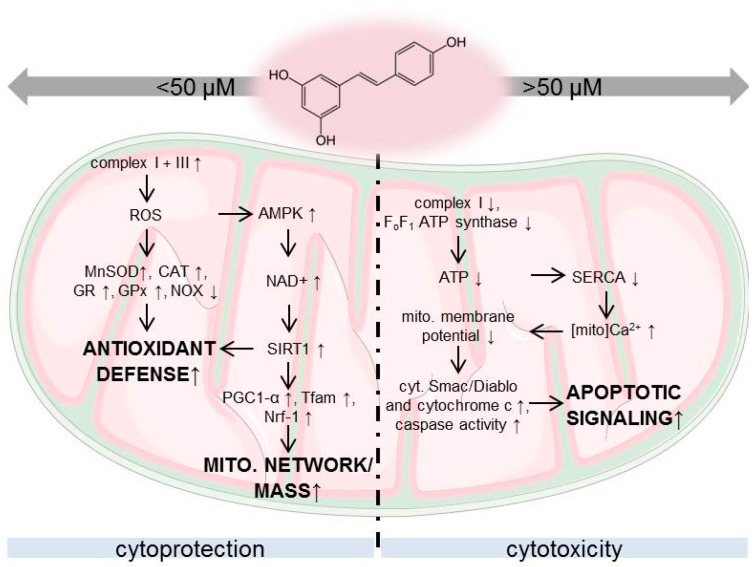
Concentration-dependent actions of RSV on mitochondrial function. AMPK: AMP-activated protein kinase, ATP: adenosine triphosphate, CAT: catalase, GPx: glutathione peroxidase, GR: glutathione reductase, MnSOD: mitochondrial superoxide dismutase 2, NAD+: nicotinamide adenine dinucleotide, NOX: NAPDH oxidase, Nrf-1: nuclear respiratory factor 1, PGC1-α: peroxisome proliferator-activated receptor-gamma coactivator 1- α, ROS: reactive oxygen species, SERCA: sarco/endoplasmic reticulum ATPase, SIRT1: sirtuin 1, Smac/ Diablo: second mitochondria-derived activator of caspase, Tfam: mitochondrial transcription factor A.

While drugs typically follow a simple concentration/effect relationship, illustrated by Paracelsus’ “Sola dosis facit venenum”, in the case of RSV, the compound exhibits duality in its therapeutic effects, depending the concentration administered: At low concentrations RSV (<50 μM) displays preferentially cytoprotective effects via initiating antioxidant defense mechanisms, AMPK/SIRT1-linked pathways or enhanced mitochondrial network formation. Oral intake of RSV of up to 25 mg/day leads to peak plasma levels of approx. 2 μM RSV and its pharmacological active metabolites, which most likely contribute to RSV’s biological activity [34,35,37]. This level may activate cytoprotective effects, like antioxidant defense mechanisms against aging-related cardiovascular damage [58] and neurodegenerative diseases [93]. At higher concentrations, RSV (>50 μM) causes cancer cell death through changes in (sub-)cellular Ca^2+^ homeostasis, disruption of mitochondrial membrane potential and activation of apoptotic caspases (Scheme 1). However, as such high peak plasma levels are hardly reached by oral administration of RSV, due to the compound’s low bioavailability [132], overcoming RSV’s low bioavailability with specific forms of therapeutic application is of particular importance, to allow full therapeutic profit from the compound’s strong in vivo anti-cancer potential. Notably, the pharmacological potential of RSV is strongly associated with mitochondria, thus highlighting the great potential of these exceptional cellular organelles as promising future drug targets.

## Figures and Tables

**Table 1:** Concentration/dose-dependent effects of resveratrol (RSV) on mitochondrial respiration as well as mitochondrial mass and structure in vitro and in vivo.

Cell Type/Tissue	Species	Effect	Conc./Dose	Incub.-Time	Reference
**mitochondrial respiration in vitro**					
HepG2		TCA cycle enzyme activity ↑, complex I activity ↑, NAD+/NADH ratio ↑	1–5 µM	48 h	Desquiret-Dumas et al., *J Biol Chem*, 2013 [47]
HepG2		complex I activity ↓	50 µM	48 h	
SW620		protein levels of OXPHOS complexes ↑, mito. respiration ↑	10 µM	48 h	Blanquer-Rosselló et al., *Biochim Biophys Acta*, 2017 [49]
fibroblasts	human (CI deficiency)	ATP ↑, ROS ↓	75 µM	48 h	Mathieu et al., *Free Radic Biol Med*, 2016 [50]
HeLa		mitochondrial ATP ↓, cytosolic ATP ↓	100 µM	3 min	Madreiter-Sokolowski et al., *Cell Physiol Biochem*, 2016 [6]
**mitochondrial respiration in vivo**					
brain (mito.)	mouse	complex I activity ↑	40 mg/kg per day *p.o.*	12 w	Gueguen et al., *PLoS One*, 2015 [48]
liver (mito.)	mouse	mitochondrial respiration ↑, NAD+/NADH ratio ↑	50mg/kg per day *p.o.*	12 w	Desquiret-Dumas et al., *J Biol Chem*, 2013 [47]
muscle	mouse	mitochondrial enzymes activity ↑, oxidative capacity ↑	10–120 ng/mL per day *p.o.*	15 w	Lagouge et al., *Cell*, 2006 [51]
liver (mito.)	rat	F_o_F_1_ ATP synthase activity ↑	nM range		Kipp et al., *Endocrine*, 2001 [52]
heart (mito.)	rat	F_o_F_1_ ATP synthase activity ↓	IC_50_ = 13–15 µM		
liver (mito.)	rat	F_o_F_1_ ATP synthase activity ↓	IC_50_ = 13–15 µM		
brain (mito.)	rat	F_o_F_1_ ATP synthase activity ↓	IC_50_ = 18.5 µM		Zheng et al., *Br J Pharmacol*, 2000 [53]
liver (mito.)	rat	F_o_F_1_ ATP synthase activity ↓	IC_50_ = 12 µM		
brain (mito.)	rat	complex III activity ↓	100 µM		Zini et al., *Drugs Exp Clin Res*, 1999 [7]
brain (mito.)	rat	ATP ↑, ROS ↓	250 mg/kg per day *p.o.*	3 w	Ghaiad et al., *Mol Neurobiol*, 2017 [54]
**mitochondrial mass and structure in vitro**					
HepG2		mito. DNA ↑, COX IV expression ↑, mitochondrial mass ↑	1 µM	12 h	Kim et al., *Antioxid Redox Signal*, 2014 [55]
GRX		MFN1 ↑ (1 µM), MFN2 ↑, OPA1 ↑, mitochondrial biogenesis ↑	1–10 µM	24 h	Meira Martins et al., *Cell Biochem Biophys*, 2015 [56]
GRX		MFN1 ↓, MFN2 ↓, OPA1 ↓, undefined cristae	50 µM	24 h	
HUVEC		SIRT1 ↑, PGC1-α ↑/ Tfam ↑/ Nrf-1 ↑, mito. DNA ↑, mitochondrial mass ↑	10 µM	48 h	Davinelli et al., *Immun Ageing*, 2013 [57]
CAEC		SIRT1 ↑, PGC1-α ↑/ Tfam ↑/ Nrf-1 ↑, mito. DNA ↑, mitochondrial mass ↑	10 µM	24 h, 48 h	Csiszar et al., *Am J Physiol Heart Circ Physiol*, 2009 [58]
SW620		SIRT3 ↑, PGC1-α ↑/ Tfam ↑/ Nrf-1 ↑, mitochondrial mass ↑	10 µM	48 h	Blanquer-Rosselló et al., *Biochim Biophys Acta*, 2017 [49]
C2C12		MFN2 ↑, mito. network ↑	10–20 µM	48 h	Robb et al., *Biochem Biophys Res Commun*, 2017 [59]
PC3					
MEF					
HMrSV5		mitophagy	50 µM		Wu et al., *Exp Cell Res*, 2016 [60]
**mitochondrial mass and structure in vivo**					
aorta	mouse (db/db)	mitochondrial biogenesis	20 mg/kg per day *p.o.*	4 w	Csiszar et al., *Am J Physiol Heart Circ Physiol*, 2009 [58]
muscle	mouse	SIRT1 ↑, PGC1-α ↑, mtDNA content ↑, size and density of mitochondria ↑	200–400 mg/kg per day *p.o.*	15 w	Lagouge et al., *Cell,* 2006 [51]

ATP: adenosine triphosphate, CI: complex I, COX IV: cyclooxygenase 4, db/db mouse: diabetic mouse model, DNA: desoxyribonucleic acid, IC_50_: half maximal inhibitory concentration, MFN1: mitofusin 1, MFN2: mitofusin 2, NAD/NADH: nicotinamide adenine dinucleotide, Nrf-1: nuclear respiratory factor 1, OPA1: optic atrophy type 1, OXPHOS: oxidative phosphorylation, PGC1-α: peroxisome proliferator-activated receptor-gamma coactivator 1-α, ROS: reactive oxygen species, SIRT1: sirtuin 1, SIRT3: sirtuin 3, TCA: tricarboxylic acid, Tfam: mitochondrial transcription factor A.

**Table 2:** Cytotoxic in vitro actions of RSV, affecting Ca^2+^ homeostasis, mitochondrial membrane potential and the mitochondrial apoptotic pathway.

Cell Type	Effect	Conc./Dose	Incub.-Time	Reference
**Ca^2+^ homeostasis in vitro**				
MCF7	ATP2A3/SERCA3 expression ↑	50 µM−200 µM	48 h, 72 h	Izquierdo-Torres et al., *Mol Carcinog*, 2017 [76]
MDA-MB-231				
MCF7	intracellular Ca^2+^ level ↑	100 µM	10–35 s	Sareen et al., *Mol Pharmacol*, 2007 [74]
MDA-MB-231				
HepG2	intracellular Ca^2+^ level ↑	100 µM	2 h, 8 h	Ma et al., *Mol Cell Biochem*, 2007 [75]
HeLa	SERCA activity ↓, mito. Ca^2+^ uptake upon IP_3_-generating stimulation ↑	100 µM	3 min	Madreiter-Sokolowski et al., *Cell Physiol Biochem*, 2016 [6]
EA.hy926		100 µM		
PC3	store-operated Ca^2+^ entry ↓, ER stress ↑	100 µM	24 h	Selvaraj et al., *Mol Carcinog*, 2016 [77]
DU145				
**mitochondrial membrane potential in vitro**				
LNCaP	membrane potential ↓	50 µM	24 h	Aziz et al., *Mol Cancer Ther*, 2006 [84]
Caki-1	membrane potential ↓	50 µM	24 h	Kim et al., *BMC Nephrol*, 2016 [80]
786-O				
TRAMP-C1, -C2, -C3	membrane potential ↓	50 µM, 100 µM	16 h	Kumar et al., *Oncotarget*, 2017 [83]
M619	membrane potential ↓	50 µM–200 µM	15 min	van Ginkel et al., *Invest Ophthalmol Vis Sci*, 2008 [78]
C918				
Mum2b				
A549	membrane potential ↓	60 µM	24 h	Gu et al., *Chem Biol Interact*, 2016 [81]
MCF7	membrane potential ↓	100 µM	10 min	Sareen et al., *Mol Pharmacol*, 2007 [74]
MDA-MB-231				
HepG2	membrane potential ↓	100 µM	10–12 h	Ma et al., *Mol Cell Biochem*, 2007 [75]
H838	membrane potential ↓	40 µg/mL ≙ 175 µM	24 h	Ma et al., *Int J Oncol*, 2015 [79]
H520		55 µg/mL ≙ 240 µM		
**mitochondrial apoptotic pathway in vitro**				
LNCaP	Bcl-2 ↓, Bax ↑	50 µM	24 h	Aziz et al., *Mol Cancer Ther*, 2006 [84]
Caki-1	activity of anti-apoptotic proteins (bcl-2, bcl-xL etc) ↓, Bax ↑, caspase-3 activity ↑	50 µM	24 h	Kim et al., *BMC Nephrol*, 2016 [80]
786-O				
TRAMP-C1, -C2, -C3	Bcl-2 ↓, Bax ↑	50 µM, 100 µM	16 h	Kumar et al., *Oncotarget,* 2017 [83]
M619	mito. Smac/Diablo release ↑, cytosolic cytochrome c ↑, caspase-3 activity ↑, caspase-9 activity ↑	50 µM–200 µM	3 h, 24 h	van Ginkel et al., *Invest Ophthalmol Vis Sci*, 2008 [78]
C918				
Mum2b				
A549	ROS ↑, caspase-3 activity ↑, cytosolic cytrochome c ↑	60 µM	24 h	Gu et al., *Chem Biol Interact*, 2016 [81]
HT-29	ROS ↑, caspase-3 activity ↑	70 µM–280 µM	24 h	Juan et al., *J Agric Food Chem*, 2008 [82]
MCF7	cytosolic cytochrome c ↑, caspase-3 activity ↑, caspase-9 activity ↑, calpain activity ↑	100 µM	24 h, 48 h, 72 h	Sareen et al., *Mol Pharmacol*, 2007 [74]
MDA-MB-231				
HepG2	mPTP opening, cytosolic cytrochome c ↑	100 µM	12 h	Ma et al., *Mol Cell Biochem*, 2007 [75]
HeLa	caspase-3 activity ↑	100 µM	3 min	Madreiter-Sokolowski et al., *Cell Physiol Biochem*, 2016 [6]
EA.hy926		100 µM		
H520	cytosolic cytrochrome c ↑, Bcl-2 ↓, Bax ↑	50–70 µg/mL ≙ 220–310 µM	24 h	Ma et al., *Int J Oncol*, 2015 [79]

ATP2A3: sarco/endoplasmic reticulum ATPase type 3, Bax: bcl-2-associated X protein, Bcl-2: B-cell lymphoma-2, Bcl-xL: B-cell lymphoma-extra large, ER: endoplasmic reticulum, IP_3_: inositol triphosphate, mPTP: mitochondrial permeability transition pore, ROS: reactive oxygen species, SERCA: sarco/endoplasmic reticulum ATPase, SERCA3: sarco/endoplasmic reticulum ATPase type 3, Smac/ Diablo: second mitochondria-derived activator of caspase.

**Table 3:** Cytoprotective in vitro and in vivo actions of RSV against toxins and disease-related cellular damage.

Cell Type/Tissue	Species	Toxic Agent/Disease	Effect	Conc./Dose	Incub.-Time	Reference
**in vitro**						
SN4741		MPP+ (50 µM)	mitochondrial dysfunction ↓	75 nM	24 h pretreatment	Zeng et al., *Neurosci Lett*, 2017 [93]
RAW 264.7		AAPH (5 mM)	AMPK activity ↑, SIRT3 expression ↑, autophagy ↑	2.5 µM	24 h co-treatment	Duan et al., *Free Radic Biol Med*, 2016 [94]
CHO-K1		beauvericin (1 µM, 5 µM)	ROS ↓	2.5 µM, 5 µM	24 h pretreatment	Mallebrera et al., *Food Chem Toxicol*, 2015 [87]
Bhas 42		benzo[a]pyrene	ROS ↓	5 µM	2 h pretreatment	Omidian et al., *Food Chem Toxical*, 2017 [88]
P12		OGD/R	MnSOD activity ↑, CAT ↑, ROS ↓, MDA ↓	10 µM	1 h pretreatment	Liu et al., *Acta Biochim Biophys Sin*, 2016 [95]
NRK-49F		high glucose (30 mM)	AMPK activity ↑, NOX4 ↓, ROS ↓	10 µM, 20 µM	1 h pretreatment	He et al., *J Mol Med*, 2016 [96]
RAW 264.7		titanium (0.1 mg/mL)	MnSOD expression ↑, Cu/ZnSOD expression ↑, GR ↑, GPx ↑, NOX ↓	10 µM, 20 µM, 40 µM	4 h pretreatment	Luo et al., *Inflammation,* 2016 [86]
HK-2		ioxitalamate (30 mg/mL)	ROS ↓	12.5 µM	48 h co-treatment	Huang et al., *Int J Mol Med*, 2016 [89]
HBE		cigarette smoke extract (3.5 %)	MFN2 ↑	20 µM	2 h pretreatment	Song et al., *PLoS One*, 2017 [72]
NRK52E		nicotine (200 µM)	MnSOD expression ↑	20 µM	24 h pretreatment	Hall et al., In Vivo, 2017 [90]
H9c2		H_2_O_2_ (500 µM)	AMPK activity ↑, ROS ↓	50 µM	30 min pretreatment	Hwang et al., *Genes Nutr*, 2008 [97]
astrocytes	rat	azide (5 mM)	MnSOD expression ↑, Cu/ZnSOD expression ↑, CAT ↑, GSH content ↑	100 µM	1 h pretreatment	Bellaver et al., *Biochim Biophys Acta*, 2016 [91]
***in vivo***						
kidney	mouse (db/db)	diabetes model	AMPK activity ↑, NOX4 ↓	40 mg/kg per day *p.o.*	12 w	He et al., *J Mol Med*, 2016 [96]
adipose tissue	mouse diabetes-induced)	diabetes model	AMPK activity ↑, mitochondrial fission ↓, ROS ↓	50 mg/kg per day *p.o.*	7 d	Li et al., *Mol Cell Endocrinol*, 2016 [73]
heart	rat (diabetes-induced)	diabetes model	SIRT1 ↑, PGC1-α ↑/ Nrf-1 ↑, ATP ↑, MnSOD expression ↑	50 mg/kg per day *p.o.*	16 w	Fang et al., *Acta Pharmacol Sin*, 2017 [98]
spinal cord	mouse (SOD1^G93^A)	ALS model	AMPK activity ↑, SIRT1 ↑	160 mg/kg per day *p.o.*	8 w	Mancuso et al., *Neurotherapeutics,* 2014 [99]
heart	mouse (diabetes-induced)	diabetes model	SERCA2a expression ↑	100 mg/kg per day *p.o.*	3 m	Sulaiman et al., *Am J Physiol Heart Circ Physiol*, 2010 [100]

AAPH: α,α′-azodiisobutyramidine dihydrochloride, ALS: amyotrophic lateral sclerosis, AMPK: AMP-activated protein kinase, ATP: adenosine triphosphate, SERCA: sarco/endoplasmic reticulum ATPase, CAT: catalase, Cu/ZnSOD: mitochondrial superoxide dismutase 1, db/db mouse: diabetic mouse model, GPx: glutathione peroxidase, GR: glutathione reductase, GSH: glutathione, H_2_O_2_: hydrogen peroxide, MFN2: mitofusin 2, MnSOD: mitochondrial superoxide dismutase 2, MPP+: 1-methyl-4-phenylpyridinium, NOX: NAPDH oxidase , NOX4: NAPDH oxidase 4, OGD/R: oxygen-glucose deprivation/reoxygenation, Nrf-1: nuclear respiratory factor 1, PGC1-α: peroxisome proliferator-activated receptor-gamma coactivator 1-α, ROS: reactive oxygen species, SIRT1: sirtuin 1, SIRT3: sirtuin 3, SOD1: superoxide dismutase 1.
